# microRNAs in Circulation Are Altered in Response to Influenza A Virus Infection in Humans

**DOI:** 10.1371/journal.pone.0076811

**Published:** 2013-10-07

**Authors:** Paul A. Tambyah, Sugunavathi Sepramaniam, Jaminah Mohamed Ali, Siaw Ching Chai, Priyadharshini Swaminathan, Arunmozhiarasi Armugam, Kandiah Jeyaseelan

**Affiliations:** 1 Department of Medicine, Centre for Translational Medicine, Yong Loo Lin School of Medicine, National University Health System, National University of Singapore, Singapore, Singapore; 2 Department of Biochemistry, Centre for Translational Medicine, Yong Loo Lin School of Medicine, National University Health System, National University of Singapore, Singapore, Singapore; 3 Department of Anatomy and Developmental Biology, School of Biomedical Sciences, Faculty of Medicine, Nursing and Health Sciences, Monash University, Clayton, Victoria, Australia; Ben-Gurion University, Israel

## Abstract

Changes in microRNA expression have been detected *in vitro* in influenza infected cells, yet little is known about them in patients. microRNA profiling was performed on whole blood of H1N1 patients to identify signature microRNAs to better understand the gene regulation involved and possibly improve diagnosis. Total RNA extracted from blood samples of influenza infected patients and healthy controls were subjected to microRNA microarray. Expression profiles of circulating microRNAs were altered and distinctly different in influenza patients. Expression of highly dysregulated microRNAs were validated using quantitative PCR. Fourteen highly dysregulated miRNAs, identified from the blood of influenza infected patients, provided a clear distinction between infected and healthy individuals. Of these, expression of miR-1260, -26a, -335*, -576-3p, -628-3p and -664 were consistently dysregulated in both whole blood and H1N1 infected cells. Potential host and viral gene targets were identified and the impact of microRNA dysregulation on the host proteome was studied. Consequences of their altered expression were extrapolated to changes in the host proteome expression. These highly dysregulated microRNAs may have crucial roles in influenza pathogenesis and are potential biomarkers of influenza.

## Introduction

Influenza A viruses cause disease in birds and a number of mammalian species including humans. The entire viral genome consists of 8 segments of negative-sense single-stranded RNA and encodes for 11 major proteins [1]. Although the life cycle of the influenza virus has been well described, the exact pathophysiology in particular, the role of microRNA in the host response to viral infection has not been well characterized. There are only a limited number of therapeutic approaches which target either the neuraminidase or the M2 ion channel. Vaccines are also limited by the seasonal antigenic drift which limits the efficacy of inactivated and live attenuated vaccines targeting specific influenza virus strains from each of the major circulating serotypes – H1N1 and H3N2 [2].

Recent studies suggest that host cellular microRNAs (miRNAs) are involved in the regulation of influenza A H1N1 replication in vitro [3-5]. miRNAs are small non-coding RNAs of approximately 22 nucleotides in length. They bind to target regions within the genome (mRNA and DNA) with perfect or partial complementarity and thus regulate the expression levels of the target gene. Since miRNAs are implicated in various cellular processes from developmental biology to disease pathology, they are believed to be powerful regulators of a range of biological processes.

Circulating miRNAs have been shown to exhibit distinctive expression patterns in relation to a number of different pathological conditions, including cerebrovascular, cardiovascular and metabolic disorders and thus have been proposed as potential biomarkers [6-8]. This role is enhanced by their remarkable stability under harsh conditions such as exposure to extreme pH, boiling and multiple freeze-thaw cycles [9]. The presence of stable circulating miRNAs also suggests a potential role as paracrine agents where a miRNA secreted by a particular organ/cell could be released into circulation to mediate its effect on a different target site. For example, miR-150 synthesized by blood and monocytic cells were packaged into microvesicles and transported to endothelial cells, effectively reducing its target gene (*MYB*) expression [10]. Thus in this study, we propose that changes in a patient’s cellular miRNA expression, due to influenza virus infection, can be detected in circulation and these profiles have the potential to aid in diagnosis and treatment.

## Methods

The study protocol was approved by the National Healthcare Group’s Domain Specific Institutional Review Board (NUS-IRB Ref Code: 09/641) and the consent procedure was approved by the Ethics committees. Written consent was obtained from the participating patients. Consenting participants contributed blood and Rayón throat/nasal swabs (Copan Diagnostics, USA) within a period of 14 days from the onset of the infection for the study and these were transported immediately to the laboratory for processing.

### Patients

In this study, a total of 23 samples from healthy volunteers and 50 samples from patients infected with influenza (43 influenza A - H1N1 and 7 influenza A - H3N2) were obtained from the National University Hospital (Singapore). Patients were hospitalized with acute respiratory tract infections and prescribed Tamiflu. No other underlying complications or infections were present. They were identified as being either infected with Influenza A H1N1 or H3N2 by the clinical laboratory using nasopharyngeal swabs by molecular diagnostic techniques using the polymerase chain reaction by the method described by Lee et al [11]. Consenting participants contributed blood and Rayón throat/nasal swabs (Copan Diagnostics, USA) within a period of 14 days from the onset of the infection for the study and these were transported immediately to the laboratory for processing. The study protocol was approved by the National Healthcare Group’s Domain Specific Institutional Review Board (NUS-IRB Ref Code: 09/641).

### Influenza virus culture

Madin Darby canine kidney cells (MDCK) (ATCC; CCL-34 ^TM^) and human lung epithelial cells (A549) (ATCC; CCL-185^TM^), maintained in Dulbecco’s Modified Eagle’s Media (DMEM) (Gibco, USA), supplemented with 10% fetal bovine serum (Gibco, USA) and antibiotics (penicillin-steptomycin) (Hyclone, USA) was used for viral culture for upper respiratory samples. Briefly, the cells were seeded onto T-25 flasks to reach 80% confluency. Prior to viral infection, the flasks were washed with PBS to remove traces of FBS. 400µl of the viral transport medium of the throat/nasal swabs diluted with 600µl of DMEM supplemented with 0.3% BSA, 2µg/ml Trypsin-TPCK (Sigma-Aldrich, St. Louis, MO) and 10ml/L of Amphotericin B (Sigma-Aldrich, St. Louis, MO) were inoculated onto T-25 flasks for a period of 1hr at 37°C with 5% CO_2_. After incubation, DMEM supplemented with 0.3% BSA, 2µg/ml Trypsin-TPCK was added and the flasks were incubated at 5% CO_2_ at 37°C for up to 96 hrs. A549 cells were supplemented with 0.2 µg/ml Trypsin-TPCK. The supernatant was saved for subsequent re-infections and exosome extraction whereas the cells were stored in Trizol reagent (Invitrogen, Calrsbad, USA) for total RNA extraction. For anti and mimic miRNA treatments the cells were seeded in 24-well plates to reach 60% confluency. 30nM of synthetic anti or miRNA mimics were transfected into the cells for a period of 48hrs following which the cells were used for infection studies as described above. The anti miRNAs and miRNA mimics used in this study are commercially available (Anti/Mimic miR-26a (MH/MC10249), Anti/Mimic miR-576-3p (MH/MC13069), Anti/Mimic miR-628-3p (MH/MC12299); Ambion, USA).

### Total RNA Isolation

Peripheral whole blood samples were collected in RNALater (Ambion, USA) and stored at -80°C. The total RNA was isolated using Ribopure™ Blood RNA isolation Kit (Ambion, USA) according to manufacturer’s protocol. Total RNA from MDCK and A549 cells stored in Trizol were also extracted according to manufacturer’s protocol. RNA concentration was determined using ND-1000 Spectrophotometer (Nanodrop™, Rockland, DE) and their integrity was verified using Agilent 2100 Bioanalyzer (Agilent Technologies, USA) and denaturing agarose gel electrophoresis. Exosomes were isolated from cell culture media using ExoQuickTM exosome precipitation kit (SystemBio, CA, USA) followed by exosomal total RNA isolation according to the manufacturer’s protocol.

### MicroRNA microarray and validation

Locked nucleic acid-modified oligonucleotide (Exiqon, Vedbaek, Denmark) probes (miRBase version 12.0) were used for the microarray. Profiling was performed only on control and H1N1 samples. Total RNA (1 µg) was 3’-end –labeled with Hy3 dye using the miRCURY LNA™ Power Labeling Kit (Exiqon) and hybridized on miRCURY LNA™ Arrays (version 12.0) according to the manufacturer’s protocol, using the MAUI® hybridization system. Hybridization images were scanned and digitized using InnoScan 700 microarray scanner (Innopsys, France). Analysis was performed using Partek® 6.6 Genomics Suite software (Copyright, Partek Inc., St Louis, MO). Briefly, background-subtracted median signal intensity of 100 was selected as a threshold value for inclusion of significantly detected miRNAs. Global sample variability was assessed by principal components analysis (PCA). First stage of normalization was carried out against a group of endogenous controls and the spike-in controls for each chip to avoid technical and experimental variations among the healthy and H1N1 infected samples. The normalized signal intensity value was log_2_ transformed and differentially regulated (H1N1 versus healthy controls) miRNAs with an absolute fold change ≥1.2 and ≤ -1.2 and *p value ≤0.05* were selected after Benjamini–Hochberg false discovery rate (FDR) correction following multiple comparisons. The FDR method was used to filter the differentially expressed miRNA. All statistical analysis was performed using the statistical tools provided by Partek® 6.6 Genomics Suite software. The results of the microarray analysis have been submitted to Gene Express Omnibus under the accession number GSE46176.

### Statistical analysis

Analysis of profiling data was performed according to previously published reports that include background subtraction, normalization, and hierarchical clustering. The data are presented as fold change; One-way ANOVA followed by Bonferroni correction was used for multiple comparisons. All statistical analysis was performed using the statistical tools provided by Partek® 6.6 Genomics Suite software. The FDR method was used to filter the differentially expressed miRNA.

### Quantitative PCR

Validation of mRNAs and miRNAs was carried out using SYBR green and TaqMan real-time PCR respectively. For mRNA quantitation 200ng of total RNA was reverse transcribed (in 10µl) using random hexamers according to the manufacturer’s protocol (Applied Biosystems, USA). Sybr green assays were performed using gene specific primers which were designed using Primer Express^TM^ (Applied Biosystems, USA). 18S rRNA was used as the endogenous calibrator for mRNA quantification. For miRNA quantitation, 10ng of total RNA was reverse transcribed (in 15µl) using specific stem-loop primers. RNUB44 was used as an endogenous calibrator. PCR reactions were performed in the Applied Biosystems 7900HT Sequence Detection System (Applied BioSystems, USA) according to the manufacturer’s protocol. All experiments (n=3) were performed in triplicates.

## Results

### Distinctive changes in miRNA transcriptome in response to influenza infection are detected in circulation

**Figure 1:**
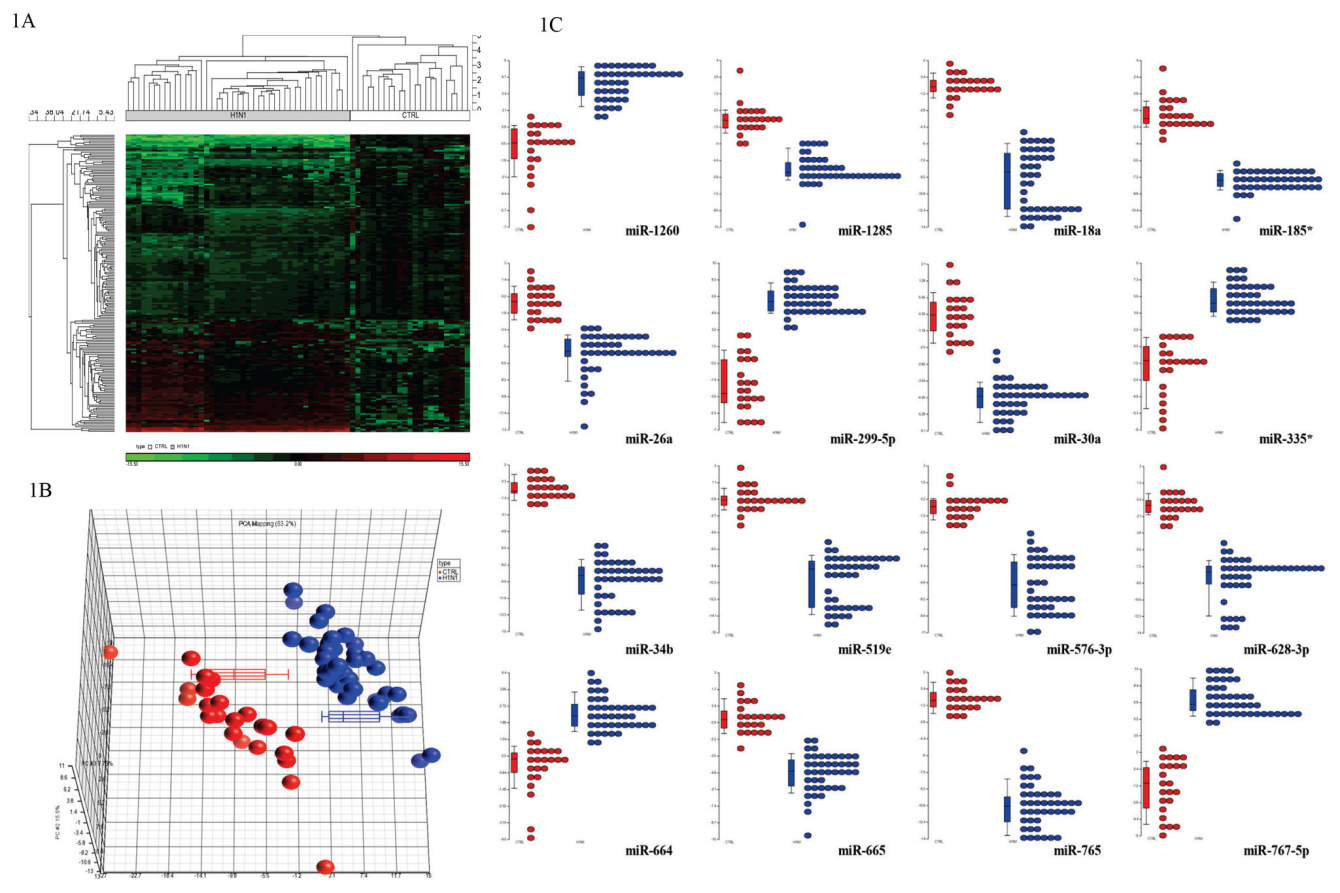
Analysis of microRNA profiles in healthy and influenza A patients. (**A**) **Hierarchical clustering of miRNA profiles**. 193 miRNAs were significantly dysregulated in influenza patients, providing a clear distinction of the pathology. The distinct clustering was seen between the miRNA profiles of the H1N1 patients and the healthy individuals. The upregulated genes are shown in red and downregulated genes are shown in green. (**B**) **Principal Component Analysis (PCA)**. PCA analysis reflected that although variation of miRNA profiles within H1N1 patients was evident, they were still clustered away from healthy individuals. Box-whisker plots also showed segregation of miRNA profiles between healthy individuals and those infected with H1N1. Red circles represent healthy individuals while blue represents H1N1 patients. (**C**) **Dot plot profiles of selected 16 miRNAs**. Distinct segregation was observed between the miRNA profiles of the H1N1 patients and the healthy individuals. Red circles represent healthy individuals while blue represents H1N1 patients.

To determine the implications of influenza viral infection on the global miRNA expression, total RNA extracted from blood samples of H1N1 infected and healthy controls were subjected to miRNA profiling. 344 conserved (in mammals) miRNAs were detected in H1N1 patients and controls. Relative expression analysis with respect to healthy individuals showed 193 miRNAs to be significantly altered in all influenza patients, regardless of the stage of infection, providing a clear distinction of the pathology (Figure 1A-B). Among them, 75 miRNAs were significantly upregulated while 118 were downregulated (Table 1). Further analysis identified 16 miRNAs (miR-1260, -1285, -18a, -185*, -299-5p, -26a, -30a, -335*, -34b, -519e, -576-3p, -628-3p, -664, -665, -765 and -767-5p) whose expression were markedly different from healthy individuals (Figure 1C). Quantitative stem-loop PCR validated the expression of 14 miRNAs (Table S1) which were subsequently selected for further studies.

**Table 1:** Significantly dysregulated circulating miRNAs in H1N1 patients.

**Significantly upregulated miRNAs in all H1N1 cases**											
**miRNA**	**p-value**	**Fold Change**	**miRNA**	**p-value**	**Fold Change**	**miRNA**	**p-value**	**Fold Change**	**miRNA**	**p-value**	**Fold Change**
miR-122*	7.05E-12	38.84	miR-210	9.32E-08	13.46	miR-491-5p	2.20E-02	2.40	miR-627	1.05E-02	3.09
miR-1224-3p	6.80E-04	3.34	miR-221*	1.94E-03	3.23	miR-492	1.81E-02	3.56	miR-630	2.41E-06	7.20
miR-1248	8.49E-07	11.29	miR-223*	1.22E-08	15.37	miR-498	2.69E-02	1.92	miR-635	2.50E-03	4.06
miR-1249	2.22E-03	2.64	miR-23a*	1.36E-07	4.97	miR-513a-3p	4.77E-05	4.01	miR-659	2.23E-02	1.91
miR-1258	1.19E-02	2.49	miR-26b*	3.71E-04	4.46	miR-513b	1.30E-04	6.01	**miR-664**	**5.36E-12**	**5.78**
miR-125a-3p	1.83E-08	10.61	miR-298	7.92E-07	6.26	miR-516b	9.61E-06	7.46	miR-675	2.76E-02	2.44
**miR-1260**	**1.93E-14**	**36.87**	**miR-299-5p**	**4.60E-19**	**363.69**	miR-525-5p	3.41E-03	2.09	**miR-767-5p**	**3.63E-20**	**319.11**
miR-1270	3.11E-03	2.79	miR-29b-1*	9.08E-07	4.13	miR-542-3p	1.54E-05	4.04	miR-874	3.62E-04	2.11
miR-1297	5.14E-03	4.54	miR-301b	9.05E-03	3.53	miR-548d-5p	1.87E-03	4.60	miR-890	4.89E-04	3.90
miR-1304	1.78E-03	2.04	miR-324-3p	2.60E-02	1.76	miR-548e	1.01E-03	3.77	miR-920	1.61E-06	5.68
miR-130b*	1.33E-03	2.60	miR-335*	**1.37E-14**	**97.72**	miR-553	4.85E-04	7.39	miR-921	9.10E-07	10.89
miR-145*	1.03E-03	3.53	miR-363*	5.79E-06	13.35	miR-556-3p	1.65E-02	3.94	miR-92a	2.83E-03	1.89
miR-146b-3p	4.70E-04	2.74	miR-374b	1.20E-02	2.39	miR-585	3.16E-03	3.54	miR-92a-2*	5.76E-04	5.27
miR-150	1.98E-02	1.60	miR-377	1.10E-03	7.12	miR-590-5p	3.99E-03	8.13	miR-934	9.63E-04	2.94
miR-152	2.17E-03	3.98	miR-377*	1.68E-07	11.35	miR-597	1.90E-02	2.86	miR-941	4.56E-13	31.85
miR-181a-2*	2.10E-04	3.05	miR-454	1.16E-07	31.75	miR-600	4.09E-03	3.09	miR-944	4.11E-04	4.40
miR-200b	6.38E-07	5.41	miR-483-5p	1.68E-03	2.70	miR-601	1.09E-03	2.50	miR-98	2.02E-08	6.93
miR-204	2.11E-03	6.36	miR-490-3p	4.16E-06	8.05	miR-618	1.12E-07	14.42	miR-99a	4.17E-03	4.56
miR-208b	3.73E-03	4.69	miR-491-3p	9.09E-03	2.38	miR-625*	4.90E-04	3.24			
**Significantly downregulated miRNAs in all H1N1 cases**											
**miRNA**	**p-value**	**Fold Change**	**miRNA**	**p-value**	**Fold Change**	**miRNA**	**p-value**	**Fold Change**	**miRNA**	**p-value**	**Fold Change**
let-7a	4.93E-09	-3.56	miR-151-3p	1.38E-04	-7.25	miR-300	4.57E-03	-2.18	miR-425	6.63E-05	-2.04
let-7a*	7.83E-03	-3.15	miR-151-5p	5.93E-03	-2.32	miR-302e	2.31E-03	-3.65	miR-425*	2.68E-06	-4.97
let-7b	1.65E-03	-2.03	miR-15b	8.54E-04	-2.28	**miR-30a**	**2.61E-19**	**-15.93**	miR-484	5.47E-08	-3.57
let-7b*	1.48E-04	-2.90	miR-16-2*	2.75E-02	-2.38	miR-30b	7.35E-12	-4.04	miR-486-5p	2.65E-02	-1.80
let-7c	1.87E-10	-5.48	miR-17	1.81E-05	-2.40	miR-30c	1.88E-06	-2.19	miR-487b	2.54E-03	-4.58
let-7d*	8.37E-08	-3.94	miR-17*	1.04E-02	-2.56	miR-30d	9.40E-07	-2.49	miR-501-5p	3.48E-06	-3.08
let-7e	2.64E-08	-3.59	miR-181a	6.05E-04	-3.37	miR-30e*	2.07E-02	-1.80	miR-519d	1.70E-08	-38.76
let-7f	1.03E-10	-6.55	miR-183	1.49E-02	-1.91	miR-32*	8.21E-06	-3.24	**miR-519e**	**9.84E-20**	**-459.31**
miR-100	1.14E-05	-11.00	miR-183*	9.79E-03	-1.85	miR-320a	1.73E-06	-2.73	miR-519e*	1.39E-02	-1.50
miR-106a	3.45E-05	-2.27	miR-185	7.16E-07	-3.48	miR-320b	2.35E-05	-2.34	miR-520d-5p	2.46E-04	-4.63
miR-106b	2.15E-04	-2.40	miR-185*	**4.45E-23**	**-81.11**	miR-320c	1.00E-05	-2.47	miR-532-5p	3.86E-03	-3.77
miR-107	1.35E-05	-2.01	miR-186	1.07E-02	-2.07	miR-320d	3.79E-05	-2.15	miR-574-3p	1.43E-03	-3.31
miR-1246	1.39E-07	-6.06	**miR-18a**	**2.96E-19**	**-446.51**	miR-324-5p	4.63E-05	-5.12	miR-574-5p	3.90E-04	-2.16
miR-125a-5p	4.52E-04	-1.82	miR-18a*	1.64E-06	-5.35	miR-331-3p	3.14E-07	-2.24	**miR-576-3p**	**4.29E-17**	**-81.03**
miR-125b	2.46E-05	-5.12	miR-18b	1.23E-08	-4.76	miR-339-3p	8.48E-04	-4.93	miR-625	8.85E-03	-1.84
miR-125b-1*	1.11E-09	-6.00	miR-1908	9.90E-09	-6.68	miR-339-5p	9.10E-11	-4.05	**miR-628-3p**	**2.28E-19**	**-144.60**
miR-125b-2*	2.82E-03	-3.57	miR-191	5.20E-06	-2.83	miR-340	8.96E-05	-3.42	miR-629	5.13E-03	-1.86
miR-1261	2.13E-06	-10.57	miR-192	2.29E-02	-3.94	miR-342-3p	2.57E-05	-3.44	miR-629*	8.91E-07	-7.01
miR-1265	1.35E-08	-4.58	miR-200c	1.79E-02	-2.87	miR-342-5p	2.14E-02	-2.26	miR-656	1.66E-02	-2.84
miR-1284	9.60E-04	-2.74	miR-208a	2.67E-03	-3.01	miR-345	2.01E-02	-3.16	**miR-665**	**1.26E-14**	**-19.87**
**miR-1285**	**3.56E-16**	**-19.96**	miR-21	2.87E-04	-5.12	**miR-34b**	**2.77E-24**	**-836.89**	miR-668	9.33E-06	-4.19
miR-1287	6.52E-03	-2.94	miR-22	3.07E-03	-2.20	miR-361-5p	1.23E-03	-2.14	miR-720	1.17E-02	-2.27
miR-1290	4.20E-04	-2.69	miR-222	2.29E-03	-2.59	miR-362-5p	1.24E-02	-2.72	miR-744	4.21E-03	-1.86
miR-1299	2.36E-02	-1.65	miR-223	1.10E-03	-1.96	miR-363	9.83E-08	-3.62	**miR-765**	**5.93E-27**	**-1503.76**
miR-130b	3.05E-07	-2.58	miR-23a	3.40E-05	-2.74	miR-375	2.47E-03	-3.60	miR-766	4.59E-03	-2.16
miR-138-2*	2.44E-03	-3.96	miR-25	1.82E-02	-1.63	miR-378	2.29E-07	-4.50	miR-92b	1.22E-06	-2.83
miR-140-3p	7.99E-07	-2.83	**miR-26a**	**1.97E-13**	**-29.66**	miR-421	2.32E-03	-4.02	miR-93	6.82E-06	-2.55
miR-146a	7.17E-04	-5.66	miR-26b	4.35E-07	-3.84	miR-422a	1.48E-03	-2.91	miR-93*	1.53E-04	-4.66
miR-148b	8.43E-05	-3.02	miR-28-5p	3.26E-09	-30.60	miR-423-3p	2.07E-09	-3.19			
miR-149*	8.26E-04	-2.33	miR-29a	5.91E-03	-2.41	miR-423-5p	8.59E-07	-3.10			

A total of 193 dysregulated miRNAs (FDR<0.05) in H1N1 are shown in this table 75 miRNAs were significantly upregulated while 118 miRNAs were downregulated in all H1N1 patients. The selected 16 miRNAs are shown in bold. * refers to the alternate miRNA strand.

### Altered expression of the selected miRNAs in Influenza A H1N1 infected cells reflects H1N1 infected blood profile

In order to confirm that the changes observed in the blood miRNA profiles were a consequence of influenza virus infection, two different cells lines, canine MDCK and human A549 were infected with throat/nasal swabs obtained from H1N1 (2009 influenza) patients. Cellular expression of all 14 miRNAs was determined using stem-loop PCR in both cell lines (Table 2). miR-1260, -26a, -335*, 576-3p, -628-3p and -664 exhibited similar expression patterns to that observed in blood. However miR-1285, -18a, -185*, -30a, -34b, -665 and -765 showed opposing expression patterns (Table 2). Thus to understand the disparities observed in miRNA expression levels in cellular and blood profiles, quantification of exosomal miRNAs was performed. Expression patterns of miRNAs packaged into exosomes and released from the cells were determined. Exosomal miR-1260 expression levels were upregulated by 50.6 ± 0.23 and 10.87 ± 0.43 fold in influenza infected A549 and MDCK cells respectively. This corresponded to the miRNA expression profiles seen in cells and circulation. miR-335* and miR-664 also showed consistently increased expression. On the contrary, the expression of downregulated miRNAs (miR-26a and miR-628-3p) differed in exosomes in comparison to cells. The differences in expression patterns of these miRNAs could be attributed to the complexities of *in vivo* events in human subjects in the case of blood profiles compared to an *in vitro* system of cell culture.

**Table 2:** Fold change of miRNA expression.

	**H1N1 infection**				
**hsa-miRNAs**	**Patient Blood**	**A549 cells**	**MDCK cells**	**A549 exosome**	**MDCK exosome**
**miR-1260**	**4.18 ± 0.19**	**1.69 ± 0.83**	**1.05 ± 0.16**	50.6 ± 0.23	10.87 ± 0.43
miR-1285	-5.16 ± 0.19	4.54 ± 0.44	-957 ± 0.81^#^	5.59 ± 0.63	NA
miR-185*	-7.57 ± 0.15	-1.19 ± 0.42	7.89 ± 0.59	NA	NA
miR-18a	-9.55 ± 0.45	-3.36 ± 0.76	3000 ± 0.12^#^	275.53 ± 0.98	305.28 ± 0.66^#^
**miR-26a**	**-5.69 ± 0.31**	**-2.28 ± 0.79**	**-6.36 ± 0.42**	153.16 ± 0.06	103.81 ± 0.43
miR-299-5p	3.60 ± 0.17	-1.21 ± 0.51	1.34 ± 0.78	197.08 ± 0.16^#^	1606.52 ± 0.22^#^
miR-30a	-4.50 ± 0.13	1.49 ± 0.83	2.45 ± 0.98	151.76 ± 0.36	2.85 ± 0.51
miR-335*	**5.27 ± 0.23**	**2.31 ± 0.30**	**14.48 ± 0.47**	1497.40 ± 0.32^#^	37.48 ± 1.99^#^
miR-34b	-9.81 ± 0.33	3.00 ± 1.43	1147 ± 1.15^#^	12.20 ± 1.21	349.37 ± 0.18^#^
**miR-576-3p**	**-6.98 ± 0.35**	**-2.49 ± 0.51**	**-203.54 ± 1.27** ^#^	NA	NA
**miR-628-3p**	**-8.48 ± 0.35**	**-2.36 ± 0.64**	**-1.60 ± 0.25**	22.05 ± 0.50	12.80 ± 0.11
**miR-664**	**2.34 ± 0.13**	**2.58 ± 0.27**	**1.89 ± 0.50**	124.97 ± 0.33	27.06 ± 0.56
miR-665	-4.82 ± 0.23	1.91 ± 0.36	- 1. 29 ± 0.69	2.17 ± 0.73	-2.21 ± 0.14
miR-765	-10.87 ± 0.32	-3.03 ± 0.57	1.13 ± 0.70	4.20 ± 0.41	12.33 ± 0.39

Expression of the 14 miRNAs dysregulated in H1N1 blood profiles, infected A549 and MDCK cells and their corresponding exosomes. miRNA cycle threshold values were normalised to endogenous control RNUB44 and expressed in relation to controls. In conditions were the miRNAs were not detected, a maximum cycle threshold value of 45 was assigned to assist in fold change calculation. miRNAs with similar expression patterns in both blood and cells are shown in bold. Data represents fold change ± SEM. * refers to the alternate miRNA strand.

# miRNA expression in controls or infected samples was undetermined. Final cycle threshold value of 45 was used to calculate the relative fold change. NA indicates miRNAs that were not detected

### Distinctions between different subtypes of influenza infections

**Figure 2:**
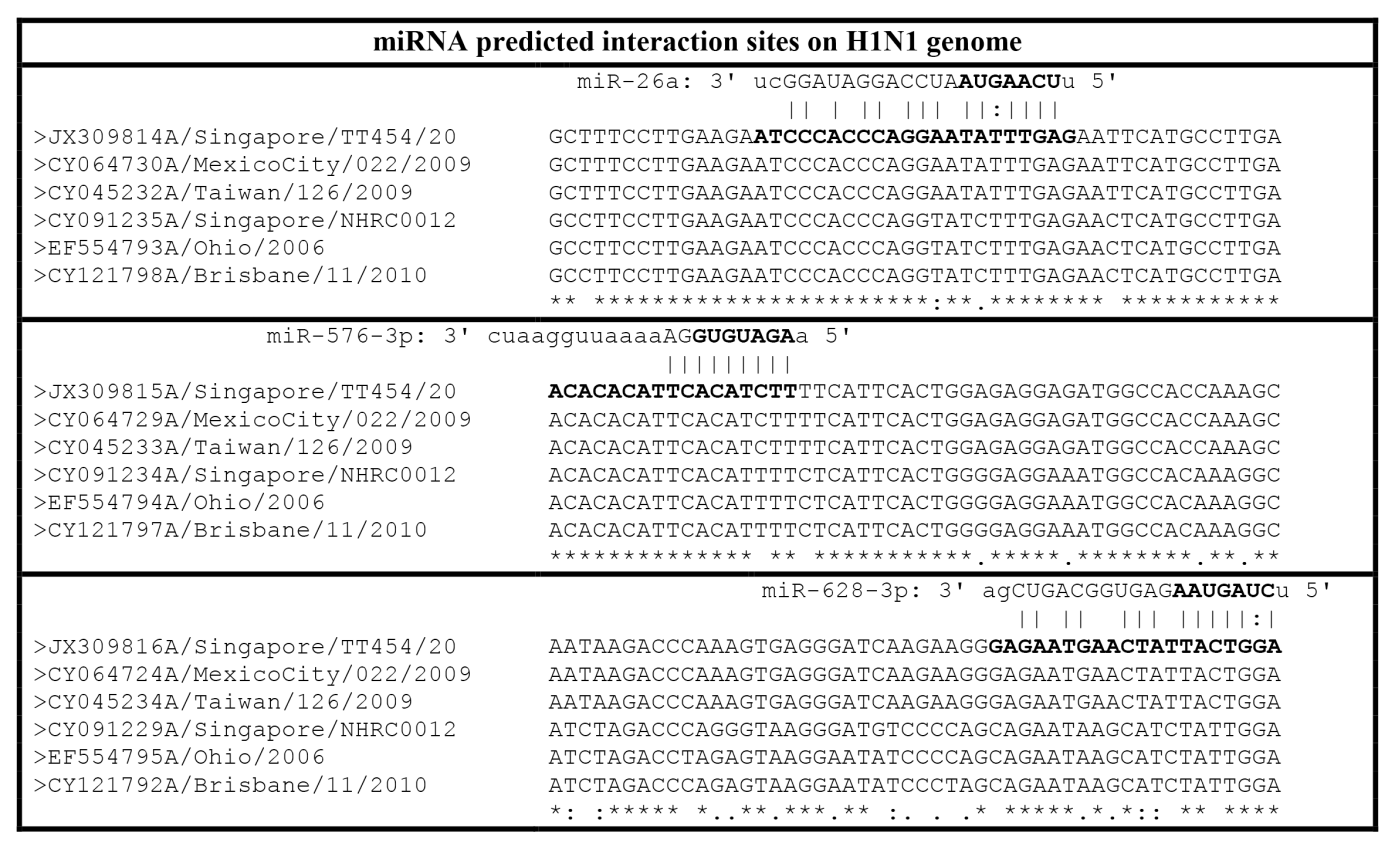
Predicted miRNA interaction sites on the H1N1 genome. The predicted miRNA interaction site in the H1N1 genome (JX309814/5/6A/Singapore/TT454/20; CY064724/29/30A/MexicoCity/022/2009; CY045232/3/4A/Taiwan/126/2009) and the miRNA seed regions are highlighted in bold. The sequences were obtained from NCBI. The corresponding accession numbers of the segments are denoted by the first eight letters/numbers in the label. The corresponding segments of H3N2 sequences (CY091229/24/35A/Singapore/NHRC0012; EF554793/4/5A/Ohio/2006; CY121792/7/8A/Brisbane/11/2010) are also shown. * refers to nucleotides which are fully conserved: indicates strong conservation within H1N1 and H3N2 whereas. indicates weak conservation within H1N1 and H3N2.

*In silico* analyses were performed using the RegRNA software [12] to map possible interactions between the miRNAs and the influenza A H1N1 and H3N2 genomes. Twelve of the miRNAs were predicted to target selected regions within the entire influenza A H1N1. The strength of their target binding to the H1N1 genome is provided in Figure S1. miR-299-5p and miR-30a did not have any predicted interaction sites with influenza A genome. Albeit the weak binding interactions, among the 12 miRNAs, miR-26a, -576-3p and -628-3p were predicted to target influenza A H1N1 only whereas the remaining 9 miRNAs were predicted to target both (H1N1 and H3N2) influenza genomes. In contrast, clustalw alignment, based on different strains (JX309814_A/ Singapore/TT454/20; CY064730A/MexicoCity/022/2009; CY045232A/Taiwan/126/2009, CY091235A/Singapore/NHRC0012; EF554793A/Ohio/ 2006; CY121798A/Brisbane/11/2010) showed that the sequences in the predicted regions for miR-26a, -576-3p and -628-3p between influenza A H1N1 and influenza A H3N2 were highly similar with single nucleotide mutations within the seed regions (Figure 2). The lack of prediction by the RegRNA program could be attributed to the differences in the seed regions due to strain specific variations.

**Figure 3:**
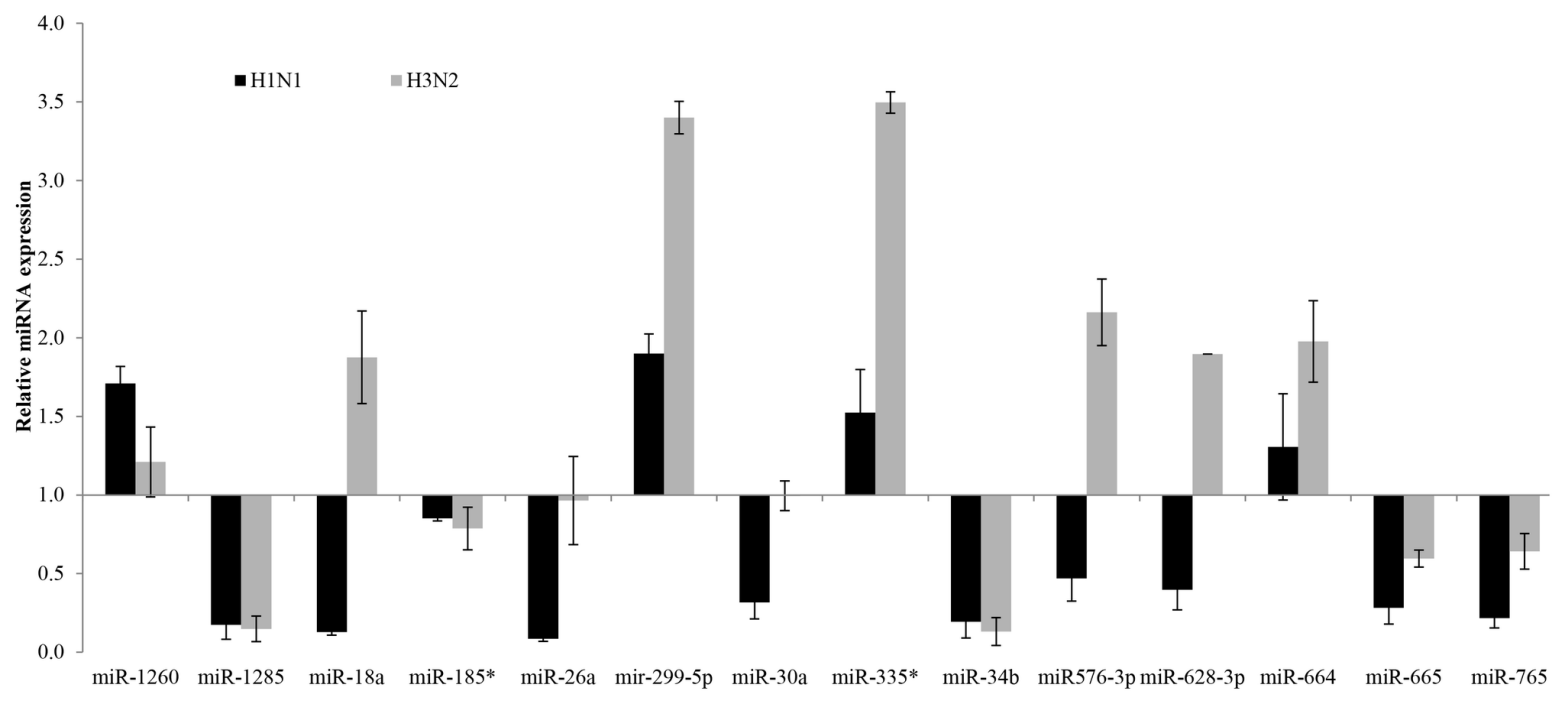
Relative miRNA expression in H1N1 and H3N2 patients. The expression of all 14 miRNAs in blood profiles of H1N1 and H3N2 patients were validated using stem-loop PCR. Relative expression values were normalised to those of healthy controls. Data shown are the mean ± SD, n≥8 for each subgroup.

Expression of all 14 miRNAs in both influenza A H1N1 and influenza A H3N2 patients were determined using stem-loop PCR. All 14 miRNAs were detected in both H1N1 and H3N2 patients. However the extent of dysregulation varied between the strains (Figure 3). These findings suggest that the panel of miRNAs might prove to be a useful diagnostic test for influenza virus infection although the extent individual miRNA expression might vary according to the strains tested.

### Identification of crucial proteins involved in influenza virus infection

**Figure 4:**
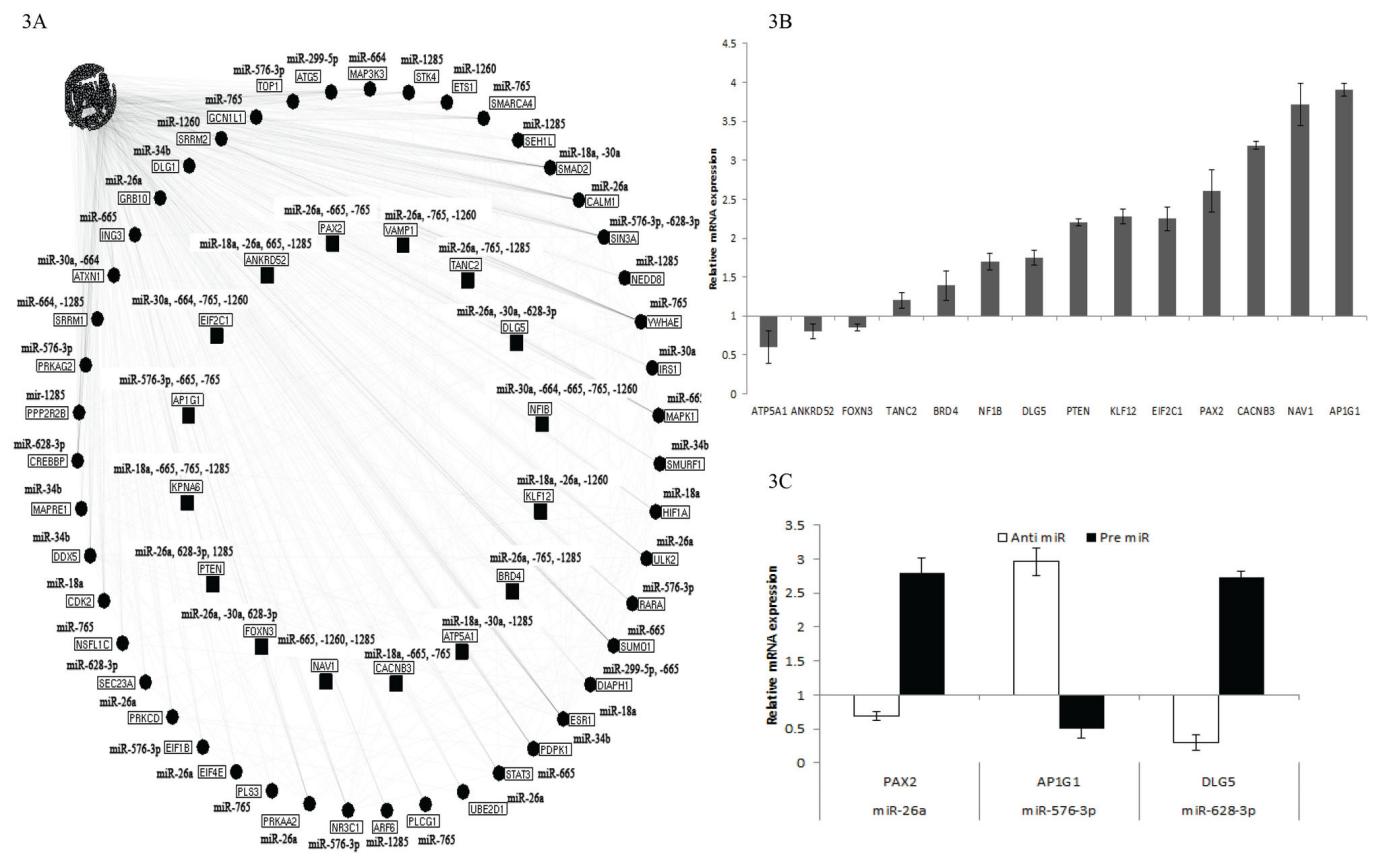
Effect of influenza infection on host genome. (**A**) **Map of Proteome interaction due to H1N1 infection**. The impact of the 14 dysregulated miRNAs on the protein expression and their corresponding interacting partners are shown here. The circle nodes represent individual proteins with high interactions (> 100) with other proteins within the system. The square nodes represent the proteins targeted by at least 3 miRNAs from the selected 14-miRNA cluster. (**B**) **mRNA expression levels in influenza infected A549 cells**. The transcript levels of the sixteen crucial proteins were determined using real-time PCR. Cycle threshold values were normalised to endogenous control 18S and expressed in relation to healthy controls. All experiments (n=3) were performed in triplicates. Data represents average of relative expression ± SEM. (**C**) **Modulation of miRNA levels**. mRNA expression levels in influenza infected A549 cells transfected with anti or pre miRNAs. The changes in the transcript levels were determined using real-time PCR. Cycle threshold values were normalised to endogenous control 18S and expressed in relation to infected A459 cells transfected with scrambled miRNAs. All experiments (n=3) were performed in triplicates. Data represents average of relative expression ± SEM.

To understand the importance of these selected miRNAs, in relation to host response, a protein interactome network was generated. Using a miRNA data integration portal [13], the predicted host targets of all the selected miRNAs were computed. Applying stringent analysis, the top 30% of the prediction targets (1121 genes) were selected (Table S2) and their protein acronyms were incorporated into a protein interaction visualizer [13]. A total of 23279 protein interactions were observed and of these 47 proteins exhibited extensive networking with others (Figure 4A). Each of them exhibited more than 100 protein to protein interactions (Table S3). Apart from these we identified 16 mRNA targets (*ANKRD52, AP1G1, ATP5A1, BRD4, CACNB3, DLG5, EIF2C1, FOXN3, KLF12, KPNA6, NAEVI, NFIB, PAX2, PTEN, TANC2 and VAMP1*) which could be potentially regulated by at least three of our selected miRNAs (Figure 4A). Quantitation of their expression in influenza infected A549 cells showed that *ANKRD52, ATP5A1, FOXN3* were downregulated whereas all the other transcripts were upregulated (Figure 4B). Expression of *VAMP1* was not detected in all cells. We studied the effects of modulating miR-26a, -576-3p and -628-3p using respective inhibitors and mimics on several of these predicted genes. Inhibition of miR-26a caused downregulation of the *PAX2* expression whereas upregulation of miR-26a increased its expression by almost 3 fold (Figure 4C). Anti miR-576-3p treatment resulted in the upregulation of *AP1G1* while pre miR-576-3p suppressed its expression. miR-628-3p was also seen to regulate *DLG5* expression. These findings suggest that the identified targets were indeed controlled by these miRNAs which are differently expressed upon influenza A infection.

## Discussion

The importance of miRNA’s regulatory potential is well recognized as dysregulation of their expression results in diverse disease phenotypes including possibly viral infections. Respiratory syncytial virus altered expression of host miRNAs (let-7f, miR-198, -24, -26b, -337-3p, -520a-5p and -595) *in vitro* eventually affecting the anti-viral host response [14] whereas Epstein-Barr virus encoded nuclear antigen 1 (EBNA1) transcriptionally activated expression of miR-127 which affected B-cell regulators [15]. We investigated the changes in the host miRNA expression due to influenza infection and for the first time, changes in circulating miRNA transcriptome in the blood of influenza A H1N1 patients are reported.

We believe that blood samples of infected patients reflect the associated pathology more accurately and thus provide a better understanding of the disease. In fact Parnell et al [16] reported that the gene expression profiles (in blood) of influenza A pneumonia was distinctly different from patients with bacterial infections or the systemic inflammatory response and that those changes in expression persisted for up to 5 days. We observed that distinct changes in the miRNA transcriptome for H1N1 patients, with no other complications, persisted for up to 14 days. These findings were then further supported by analyzing selected miRNA expression patterns in influenza infected cell lines.

Profiling of H1N1 infected blood samples identified a cluster of 193 miRNAs to be significantly altered. The altered blood miRNA profiles were similar to several reports by various groups studying influenza virus infection *in vitro* (Table S4) [4,5,17]. Several of these miRNAs were found to be conserved and similarly altered in infected MDCK cells as well. let-7c, miR-146a, -200c, -23a, -29a, -361-5p were some of those that have been reported in relation to influenza infection from cell culture systems. let-7c was identified as a regulator of the M1 segment of the influenza virus. let-7c downregulated M1 expression at both the cRNA and protein levels while, transfection with let-7c inhibitors enhanced the expression of M1 [5]. Downregulation of miR-23a was seen in other studies as well. miR-23a was identified to be a regulator of Nedd4L, a ubiquitin ligase [18]. Nedd4L regulates the TGF-β pathway by inducing proteasome-dependent degradation of the TGF-β type 1 receptor and its mediator Smad 2/3 [19]. miR-23a repression was proposed to indirectly suppress TGF-β signaling and contribute to disease persistence [18]. Analysis based on the 193 miRNAs also showed that the TGF-β signaling was one of the top 5 pathways to be affected in influenza infection (Table S5).

TNFα and interleukin-6 (IL-6) are components of the innate immune response. IL-6 is an important cytokine that orchestrates the acute-phase response to inflammation and actually contributes to the febrile response to influenza and other infections, via phosphorylation of STAT3 (signal transducer and activator of transcription 3). miR-18a, -146a and -223 which were detected in the blood miRNA profiles, are all implicated in maintaining the fine balance of the *IL-6* expression as these miRNAs target various molecules along the IL-6 pathway: miR-18a targets *PIAS3*, an inhibitor of STAT3 [20]. Repression of miR-18a suggested increased levels of the inhibitor. However repression of miR-223 a direct target of *STAT3* was also seen in our patients indicating upregulation of *STAT3* expression [21]. Furthermore miR-146a, regulator of *IL-6* was also suppressed [22]. These findings indicate the existence of a complex feedback system between the miRNAs in the regulation of the acute response due to influenza infection in patients with moderately severe influenza infection who are hospitalized with fever and symptoms.

We found 14 miRNAs which showed distinct segregation and extreme dysregulation in influenza patients for up to 2 weeks (acute infection until recovery) of follow up suggesting that these changes were not solely attributed to an acute inflammatory response driven by interferons or cytokines. Influenza infection induced expression of primary miRNAs rather than enhancing precursor miRNA processing and the changes in expression were far more potent than those triggered by anti-viral molecules such as interferons, interleukin-6 and TNFα in human respiratory cells [17]. TNFα adminstration increased miR-146a expression by 14.3 fold which further increased to 150 fold in response to influenza A infection [17]. Corroborating these observations, we noticed that the 14 miRNAs selected for further studies exhibited extreme dysregulation in influenza infected patients. miR-299-5p and -335* were the 2 most highly expressed miRNAs in influenza patients (Table 1). In contrast, miR-765, -34b, -519e, -18a, -628-3p, -185*, -576-3p, -519d, -28-5p, -26a, -1285, -665 and -30a were the 13 most down-regulated miRNAs in H1N1 patients. Except miR-519d and -28-5p, all the other 11 miRNAs belonged to the 14 miRNA cluster. These findings imply that indeed the 14 miRNAs shortlisted for further studies were highly crucial in H1N1 infection.

Among these upregulated miRNAs, miR-1260, -335* and -664 consistently exhibited similar expression patterns in blood, cells and exosomes in response to influenza infection. On the contrary downregulated miRNAs-26a, -576-3p and -628-3p showed similar expression patterns in blood and infected cells only. miR-576-3p was not detectable in exosomes, while miR-26a and miR-628-3p were upregulated in exosomes. Though it is not clear as to what causes this discrepancy in cellular and exosomal expression patterns, it has been previously shown that miRNAs upregulated in aortic aneurysm tissue were downregulated in the patients’ circulation suggesting different regulatory mechanisms at play [23]. Thus further studies are needed to determine the cause of the differential miRNA expression in cellular and exosomal levels.

Our protein interactome analysis enabled a better understanding of the implications of the altered miRNA expression in the host genome. Forty-seven targets that had extensive protein to protein interactions and their direct interactions to the altered miRNAs were identified (Figure 4A). Majority of them are implicated in genome maintenance and cellular fate and organization (Table S3). *MAPK1, CREBBP, PRKCD, PRKAG2, SUMO1, NEDD8, DLG1* and *ATXN1* were some of the targets identified to be regulated by this cluster of altered miRNAs. *NEDD8*, an ubiquitin like protein, has been implicated in viral replication in herpesviruses. These viruses have a cysteine protease which functions as a NEDD8-specific deneddylase leading to the deregulation of cell cycle and the establishment of an S-phase-like environment which is required for efficient replication of the viral genome [24]. Expression of *NEDD8* was increased in influenza infected cells suggesting a defense mechanism. To date, to our knowledge, this phenomenon has not been described in influenza.

Konig et al [25] identified 295 human host factors which were considered important for influenza viral replication and among these *MAPK1, CREBBP, PRKCD, PRKAG2*, and *SUMO1* were also reported, confirming the robustness of our study. *MAPK1* is implicated in the PKC and PI3k/AKT pathways which are crucial for viral replication. The P13K/Akt survival pathway is postulated to play crucial roles in influenza A infection. Inhibition of PI3K prevents virus uptake into endosomes [26]. Akt/PKB is a major PI3K effector which phosphorylates downstream targets such as caspase-9, BAD and glycogen synthase kinase 3β. This leads to the suppression of apoptosis and promotes survival and proliferation eventually preventing premature cell death and allowing time for viral propagation. *CREBBP, PRKCD* are implicated in MAPK signaling which is crucial for viral protein production and export while *PRKAG2* is implicated in post-entry processes and autophagy in influenza infection [25]. Increased expression of DLG1 protein was reported in influenza infection and interaction of the viral NS1 segment with DLG1 has been implicated in tight junction disruption [27]. This disruption is proposed to be in favour of viral replication where it is thought to enhance the dissemination of the virus throughout the host. Interaction of ATXN10 with the influenza A M2 protein has also been reported [28] yet our analysis suggests ATXN1 might also be an important protein in influenza A infection.

Apart from these 47 targets, our study also highlighted 16 targets (*ANKRD52, AP1G1, ATP5A1, BRD4, CACNB3, DLG5, EIF2C1, FOXN3, KLF12, KPNA6, NAEVI, NFIB, PAX2, PTEN, TANC2 and VAMP1*) that were predicted to be regulated by more than one of our selected miRNAs. Increased expression of *PAX2* gene and protein was associated with enhanced resistance to apoptosis in Kaposi sarcoma–associated herpesvirus infected cells [29]. We observed PAX2, a transcription factor, to be significantly upregulated upon influenza infection and its expression could be modulated by regulating miR-26a. Interestingly anti miR-26a suppressed *PAX2* expression suggesting that miR-26a is crucial for *PAX2* expression. This observation was further strengthened when an increase in *PAX2* expression was seen in increased miR-26a levels. These findings suggest that miR-26a plays a crucial role in activating *PAX2*. *AP1G1* (AP-1 complex subunit gamma-1) encodes for one of the subunits of adaptins which are important components of clathrin-coated vesicles [30]. One of the ways which the Influenza virus enters the host cells is via clathrin-mediated endocytosis, thus regulating *AP1G1* expression with miR-576-3p could affect the viral entry into cells for we observed miR-576-3p to be a repressor of *AP1G1* expression. DLG5 was also identified as a crucial host protein for knockdown of its gene affected the influenza virus lifecycle [25]. Correspondingly, we also observed an increase in *DLG5* expression upon influenza infection and we show that *DLG5* could be regulated by miR-628-3p. Similar to miR-26a:*PAX2*, miR-628-3p was observed to be an activator of *DLG5* expression. miRNA based post-transcriptional activation has been observed in other studies as well [31]. Our findings supported with existing data, highlight that the selected cluster of miRNAs are indeed regulating crucial targets of relevance to influenza infection.

While many groups have demonstrated the presence of miRNA changes *in vitro* using cell culture systems [4,5,17], we believe that we are the first to present changes in the circulating miRNA transcriptome in the blood of patients with acute influenza infection which was severe enough to warrant hospitalization of the patients. The findings of this study highlight the fact that the blood miRNA transcriptome (as opposed to respiratory tract samples which are usually studied) reveals important information about influenza infection and the corresponding host response. Further studies are clearly needed to explore the relative contribution of these selected miRNAs. Apart from regulating crucial genes in the host genome, this cluster of 14 miRNAs is also predicted to target the viral genome. Although weak binding interaction were seen and their relevance to regulating viral transcripts is yet to be established miR-26a, -576-3p and -628-3p were shown to be specific for H1N1. Thus these miRNAs represent potential targets which could be used as potential therapeutics for treatment against influenza A. With miR-122 being tested in phase 2 clinical trials against Hepatitis C (http://www.santaris.com), it is possible that some of these miRNAs could be potential therapeutic agents to expand our limited armamentarium against influenza.

## Conclusion(S)

We have shown that changes occurring in the miRNA transcriptome in response to influenza infection can be detected in hospitalized patients’ blood samples. Dysregulation in a cluster of 14 miRNAs was shown to have vast impact on the host proteome expression. This has the potential for a major impact on diagnostics and therapeutics for influenza.

## Supporting Information

Figure S1 **Strength of target binding for selected miRNAs against H1N1 genome.**
(DOC)

Table S1 **Validation of selected miRNAs.**
(DOC)

Table S2 **Top 30% of gene targets regulated by the selected miRNAs.**
(XLS)

Table S3 **Compilation of proteins exhibiting extensive associations.**
(DOC)

Table S4 **Tabulation of miRNAs reported in other in vitro Influenza studies.**
(XLS)

Table S5 **Top 10 pathways affected by the 193 dysregulated miRNAs.**
(DOC)
