# Supplementary material for: microRNAs in Circulation Are Altered in Response to Influenza A Virus Infection in Humans
Source: PLoS One. 2013 Oct 7;8(10):e76811. doi: 10.1371/journal.pone.0076811 (PMC3792094; doi:10.1371/journal.pone.0076811)
Supplement: Figure S1 — Strength of target binding for selected miRNAs against H1N1 genome. (DOC) [file pone.0076811.s001.doc]

**Figure S1: Strength of target binding for selected miRNAs against H1N1 genome.**

Values given are in minimum free energy (kcal/mol)
