# Supplementary material for: microRNAs in Circulation Are Altered in Response to Influenza A Virus Infection in Humans
Source: PLoS One. 2013 Oct 7;8(10):e76811. doi: 10.1371/journal.pone.0076811 (PMC3792094; doi:10.1371/journal.pone.0076811)
Supplement: Table S1 — Validation of selected miRNAs. (DOC) [file pone.0076811.s002.doc]

**Table S1: Validation of selected miRNAs.**

Expression of selected 16 miRNAs was determined via stem-loop PCR.

| **miRNA** | **Ct values** |
| --- | --- |
| **miR-1260** | 23.29 ± 0.37 |
| **miR-1285** | 29.55 ± 0.15 |
| **miR-18a** | 21.23 ± 0.13 |
| **miR-185*** | 27.11 ± 0.26 |
| **miR-26a** | 19.40 ± 0.25 |
| **miR-299-5p** | 32.37± 0.21 |
| **miR-30a** | 19.67 ± 0.17 |
| **miR-335*** | 29.96 ± 0.10 |
| **miR-34b** | 33.08 ± 0.44 |
| **miR-519e** | Undetermined |
| **miR-576-3p** | 29.76 ± 0.02 |
| **miR-628-3p** | 27.29 ± 0.39 |
| **miR-664** | 25.07 ± 0.28 |
| **miR-665** | 30.16 ± 0.06 |
| **miR-765** | 32.97 ± 0.40 |
| **miR-767-5p** | Undetermined |
