# Supplementary material for: microRNAs in Circulation Are Altered in Response to Influenza A Virus Infection in Humans
Source: PLoS One. 2013 Oct 7;8(10):e76811. doi: 10.1371/journal.pone.0076811 (PMC3792094; doi:10.1371/journal.pone.0076811)
Supplement: Table S3 — Compilation of proteins exhibiting extensive associations. (DOC) [file pone.0076811.s004.doc]

**Table S3: Compilation of proteins exhibiting extensive associations.**

| **Gene Name** | **Protein ID** | **Number of associations** | **Ontology** |
| --- | --- | --- | --- |
| **ARF6** | P62330 | 153 | Cellular fate and organisation |
| **ATG5** | Q9H1Y0 | 444 | Cellular fate and organisation |
| **ATXN1** | P54253 | 197 | Transcription |
| **CALM1** | P62158 | 434 | Genome maintenance |
| **CDK2** | P24941 | 139 | Genome maintenance |
| **CREBBP** | Q92793 | 320 | Transcription |
| **DDX5** | P17844 | 217 | Transcription |
| **DIAPH1** | O60610 | 164 | Cellular fate and organisation |
| **DLG1** | Q12959 | 109 | Genome maintenance |
| **EIF1B** | O60739 | 162 | Translation |
| **EIF4E** | P06730 | 219 | Genome maintenance |
| **ESR1** | P03372 | 579 | Genome maintenance |
| **EST1** | P14921 | 100 | Genome maintenance |
| **GCN1L1** | Q92616 | 255 | Translation |
| **GRB10** | Q13322 | 177 | Cellular fate and organisation |
| **HIF1A** | Q16665 | 300 | Cellular fate and organisation |
| **ING3** | Q9NXR8 | 165 | Transcription |
| **IRS1** | P35568 | 117 | Cellular fate and organisation |
| **MAP3K3** | Q99759 | 187 | Cellular fate and organisation |
| **MAPK1** | P28482 | 355 | Genome maintenance |
| **MAPRE1** | Q15691 | 160 | Genome maintenance |
| **NEDD8** | Q15843 | 117 | Protein fate |
| **NR3C1** | P04150 | 199 | Transcription |
| **NSFL1C** | Q9UNZ2 | 115 | Unknown |
| **PDPK1** | O15530 | 414 | Cellular fate and organisation |
| **PLCG1** | P19174 | 233 | Cellular fate and organisation |
| **PLS3** | P13797 | 117 | Cellular fate and organisation |
| **PPP2R2B** | Q00005 | 182 | Cellular fate and organisation |
| **PRKAA2** | P54646 | 395 | Genome maintenance |
| **PRKAG2** | Q9UGJO | 435 | Genome maintenance |
| **PRKCD** | Q05655 | 131 | Cellular fate and organisation |
| **RARA** | P10276 | 115 | Genome maintenance |
| **SEC23A** | Q15436 | 140 | Protein fate |
| **SEH1L** | Q96EE3 | 123 | Genome maintenance |
| **SIN3A** | Q96ST3 | 352 | Unknown |
| **SMAD2** | Q15796 | 375 | Cellular fate and organisation |
| **SMARCA4** | P51532 | 252 | Genome maintenance |
| **SMURF1** | Q9HCE7 | 116 | Cellular fate and organisation |
| **SRRM1** | Q8IYB3 | 106 | Transcription |
| **SRRM2** | Q9UQ35 | 141 | Transcription |
| **STAT3** | P40763 | 164 | Cellular fate and organisation |
| **STK4** | Q13043 | 228 | Cellular fate and organisation |
| **SUMO1** | P63165 | 438 | Genome maintenance |
| **TOP1** | P11387 | 222 | Genome maintenance |
| **UBE2D1** | P51668 | 127 | Genome maintenance |
| **ULK2** | Q8IYT8 | 313 | Cellular fate and organisation |
| **YWHAE** | P62258 | 472 | Genome maintenance |
