# Supplementary material for: microRNAs in Circulation Are Altered in Response to Influenza A Virus Infection in Humans
Source: PLoS One. 2013 Oct 7;8(10):e76811. doi: 10.1371/journal.pone.0076811 (PMC3792094; doi:10.1371/journal.pone.0076811)
Supplement: Table S5 — Top 10 pathways affected by the 193 dysregulated miRNAs. (DOC) [file pone.0076811.s006.doc]

**Table S5: Top 10 pathways affected by the 193 dysregulated miRNAs**

| **Pathways** | **-ln(p-value)** | | **Affected genes** |
| --- | --- | --- | --- |
| **ECM-receptor interaction** | 37.98 | *COL6A3, FNDC5, COL4A6, COL5A1, COL1A1, LAMA2, COL3A1, DAG1, ITGA2, ITGA6, COL4A4, COL5A3, HSPG2, COL5A2, COL1A2, COL2A1, LAMC1, COL4A1, COL11A1* | |
| **Ribosome** | 34.19 | *RPL37A, RPL37* | |
| **TGF-beta signaling pathway** | 34.14 | *RBL2, CDKN2B, IFNG, TGFBR1, ID2, ID4, LTBP1, RHOA, ROCK1,* ***SMURF1****, ID3, SMAD7,* ***SMAD2****, SMAD5, SKP1A, ACVR2A, INHBB,* ***MAPK1****, THBS1, CREBBP, BMP2, ACVR2B, SMURF2, ZFYVE16, BMP4, BMPR2, PITX2, RPS6KB1, SMAD4, BMPR1A* | |
| **Focal adhesion** | 34.09 | *COL6A3, MAPK8, COL4A6, COL5A1, CAV2, PDGFB, COL1A1, LAMA2, COL3A1, ITGA2, ITGA6, PIK3R1, COL4A4, COL5A3, VEGFA, PTEN,PTENP1, CDC42, COL5A2, AKT2, PIK3R2, COL1A2, CCND2, COL2A1, LAMC1, IGF1, PDGFRB, COL4A1, AKT3, PDGFC, COL11A1* | |
| **Methionine metabolism** | 27.11 | *MTAP, TRDMT1* | |
| **Heparan sulfate biosynthesis** | 24.43 | *HS3ST3B1, HS3ST2* | |
| **Wnt signaling pathway** | 23.95 | *FZD7, CXXC4, MAPK8, PRKCA, LEF1, WNT3, WNT16, LRP6, TBL1X,* ***SMAD2****, FZD3, PRKX, APC, DAAM1, VANGL1, RAC1,* ***CREBBP****, FBXW11, PPP2CA, SIAH1, TCF7L2, PPP3CA, NLK, FZD1, PLCB1, DKK2, FZD4, NFAT5, PRICKLE2, EP300, JUN, CTBP2, CCND1, SMAD4, CTNNB1* | |
| **Adherens junction** | 19.79 | *PTPRM, LEF1, TGFBR1, IGF1R, SNAI1, ENSG00000158195,* ***SMAD2****, SORBS1, MAPK1, RAC1, INSR,* ***CREBBP****, CDC42, TCF7L2, SSX2IP, NLK, EP300, LMO7, IQGAP1, SMAD4, CTNNB1* | |
| **Cell Communication** | 18.04 | *DSG1, GJA7* | |
| **mTOR signaling pathway** | 16.32 | *PDPK1,* ***MAPK1****, RPS6KA3* | |
